# Phosphoproteomic Analysis of Breast Cancer-Derived Small Extracellular Vesicles Reveals Disease-Specific Phosphorylated Enzymes

**DOI:** 10.3390/biomedicines10020408

**Published:** 2022-02-09

**Authors:** Zoran Minic, Nico Hüttmann, Suttinee Poolsup, Yingxi Li, Vanessa Susevski, Emil Zaripov, Maxim V. Berezovski

**Affiliations:** 1 John L. Holmes Mass Spectrometry Facility, Faculty of Science, University of Ottawa, Ottawa, ON K1N 6N5, Canada; 2 Department of Chemistry and Biomolecular Sciences, University of Ottawa, Ottawa, ON K1N 6N5, Canada; nhutt069@uottawa.ca (N.H.); spool093@uottawa.ca (S.P.); yli840@uottawa.ca (Y.L.); vsuse027@uottawa.ca (V.S.); ezari068@uottawa.ca (E.Z.)

**Keywords:** breast cancer, phosphoproteomics, small extracellular vesicles, ATP citrate lyase (ACLY), phosphofructokinase-M (PFKM), sirtuin-1 (SIRT1), sirtuin-6 (SIRT6)

## Abstract

Small membrane-derived extracellular vesicles have been proposed as participating in several cancer diseases, including breast cancer (BC). We performed a phosphoproteomic analysis of breast cancer-derived small extracellular vesicles (sEVs) to provide insight into the molecular and cellular regulatory mechanisms important for breast cancer tumor progression and metastasis. We examined three cell line models for breast cancer: MCF10A (non-malignant), MCF7 (estrogen and progesterone receptor-positive, metastatic), and MDA-MB-231 (triple-negative, highly metastatic). To obtain a comprehensive overview of the sEV phosphoproteome derived from each cell line, effective phosphopeptide enrichment techniques IMAC and TiO_2_, followed by LC-MS/MS, were performed. The phosphoproteome was profiled to a depth of 2003 phosphopeptides, of which 207, 854, and 1335 were identified in MCF10A, MCF7, and MDA-MB-231 cell lines, respectively. Furthermore, 2450 phosphorylation sites were mapped to 855 distinct proteins, covering a wide range of functions. The identified proteins are associated with several diseases, mostly related to cancer. Among the phosphoproteins, we validated four enzymes associated with cancer and present only in sEVs isolated from MCF7 and MDA-MB-231 cell lines: ATP citrate lyase (ACLY), phosphofructokinase-M (PFKM), sirtuin-1 (SIRT1), and sirtuin-6 (SIRT6). With the exception of PFKM, the specific activity of these enzymes was significantly higher in MDA-MB-231 when compared with MCF10A-derived sEVs. This study demonstrates that sEVs contain functional metabolic enzymes that could be further explored for their potential use in early BC diagnostic and therapeutic applications.

## 1. Introduction

Extracellular vesicles (EVs) derived from human cancer cell lines are involved in multiple biological processes in tumor biology, such as modulation of the microenvironment, angiogenesis, sustained growth, and tissue invasion [1,2,3,4,5,6,7]. EVs act as transport vectors or signal transducers that can deliver specific biological information by transferring bioactive content (nucleic acids, proteins, and metabolites) from donor to nearby or distant cells [8,9,10]. Therefore, EVs emerged as key regulators of cell–cell communication within multicellular organisms, in health and disease. EVs include several subtypes of membrane-bound vesicles, including exosomes and microvesicles, distinguished by their biogenesis pathway. These subtypes may be roughly categorized by their measured diameter, with small extracellular vesicles (sEVs) or exosomes in the range of 30 to 150 nm [11,12] and medium extracellular vesicles (mEVs) in the range of 50 nm to 1000 nm in diameter [13], this study’s vesicles with a measured diameter in the range of 50 to 300 nm are referred to as sEVs.

In order to elucidate the molecular changes that coincide with cancer or discover new biomarkers, it is important to measure protein expression in EVs, followed by the identification of post-translationally modified proteins such as phosphorylated proteins. Protein phosphorylation is the most important post-translational modification that dominates signaling transduction, which plays an essential role for almost all cellular functions and is involved in major regulatory mechanisms of cell signaling networks [14]. Recent studies have indicated that certain phosphoproteins encapsulated within EVs function as key regulators in the tumor microenvironment [15,16]. A recent study found high expression levels of tyrosine kinases (RTKs), such as phosphorylated EGFR and HER2, in MCF7 cell line-derived exosomes; these sEVs were capable of stimulating the MAPK pathway in monocytes through the transport of functional RTKs, leading to the inactivation of apoptosis-related caspases [17]. It has been reported that human colorectal cancer exosomes, derived from the SW620 cell line, were found to contain 313 phosphoproteins with 1091 phosphosites, of which 202 were newly discovered [16]. These exosome-derived phosphoproteins had a remarkably high level of tyrosine-phosphorylated proteins (6.4%) which were functionally relevant to ephrin signalling pathway-directed cytoskeleton remodeling.

Some studies reported the detection of phosphoproteins in biofluids for disease diagnosis [18,19,20]. For instance, an in-depth analysis of phosphoproteomes in plasma from both microvesicles and exosomes measured phosphorylation changes across patients with breast cancer (BC) and healthy individuals [18]. Using label-free quantitative phosphoproteomics, 144 phosphoproteins were identified in plasma EVs, which were more abundantly expressed in patients diagnosed with breast cancer compared with healthy controls [18]. In another quantitative EV phosphoproteomic study, plasma samples from patients diagnosed with kidney cancer were analyzed in order to identify direct markers of cellular signaling and disease progression [19]. The results of these analyses revealed 28 proteins present in kidney cancer samples that were not detected in the control samples. Several EV isolation methods have been assessed for highly efficient capture of EVs from human urine samples [20]. For example, using the EVTRAP isolation method, close to 2000 unique phosphopeptides were identified from 10 mL of urine. Data obtained for protein phosphorylation in EVs presents a potential opportunity for understanding cancer signaling and early-stage cancer diagnosis.

Cell lines are commonly used as models in cancer biology since they are easily grown and relatively inexpensive, making them suitable for high-throughput testing and omics studies [21]. Blood is a systemic source of EVs from all tissue types in the body, not only breast tumors; therefore, it is difficult to select appropriate biomarkers relevant to breast cancer (BC). Comparative analyses of body fluids and cell lines may help find more tissue specific biomarkers. In recently published work, the difference in proteomes between cancerous (MCF7 and MDA-MB-231) and noncancerous (MCF10A) cell line-derived EVs has been investigated [6,7]. The results revealed that 87 sEV [6] and 112 mEV [7] proteins relevant to BC in which MDA-MB-231 cell line-derived sEV proteins were proposed as potential breast cancer biomarkers for disease diagnosis and prognosis as well as for potential therapeutic targets and resistance against chemotherapy agents. Moreover, the study also validated three enzymes—ornithine aminotransferase (OAT), transaldolase (TALDO1) and bleomycin hydrolase (BLMH)—using standard enzymatic assays. Two of the three enzymes, OAT and TALDO1, had significantly higher specific enzymatic activities in MDA-MB-231-derived sEVs than MCF10A. BLMH was found to be highly expressed in MDA-MB-231 microvesicles (MVs) when compared with MCF10A-derived MVs. EV enzymes have not been previously investigated in a comprehensive manner. Therefore, this study aimed to provide support for the investigation of enzymes derived from EVs for BC diagnosis and therapeutic applications.

The aim of this study was to gain insight into the phosphoproteome of sEVs derived from the metastatic BC cell lines MDA-MB-231 and MCF7 and from the non-cancerous cell line MCF10A. Differences in phosphoproteomes were observed between the control cell line, MCF10A, and BC cell lines, MDA-MB-231 and MCF7. We selected and validated four phosphoenzymes: ATP citrate lyase (ACLY), phosphofructokinase-M (PFKM), sirtuin-1 (SIRT1), and sirtuin-6 (SIRT6) identified in both MDA-MB-231 and MCF7 that might be potentially relevant to BC diagnosis.

## 2. Materials and Methods

### 2.1. Cell Culturing and sEV Isolation

Breast cancer epithelial cell lines MDA-MB-231 (ATCC HTB-26) and MCF7 (ATCC HTB-22) and the non-tumorigenic breast epithelial cell line MCF10A (ATCC CRL-10317) were obtained from the American Type Culture Collection (ATCC) and used in this study. MDA-MB-231 and MCF10A cells were cultured as described in our previous work [6]. MCF7 cells were cultured in EV-depleted DMEM/Ham’s F12 (GIBCO-Invitrogen) supplemented with 10% fetal bovine serum (Sigma Life Science, St. Louis, USA). The three cell lines were plated in increased attachment cell culture dishes (VWR) and grown for 7 days. Each liter of cell culture supernatant was harvested from approximately 4 × 10^8^ cells.

### 2.2. Differential Ultracentrifugation (UC)

Small EVs were isolated from cell culture supernatant by UC, as described previously [6]. Conditioned medium (240 mL) was harvested after 7 days of incubation and immediately centrifuged at 300× *g* for 10 min. Apoptotic bodies were removed by centrifugation at 2000× *g* using a Sigma13190 rotor (MBI) for 20 min. Samples were then spun at 16,500× *g* for 1 h using an SW28 Ti rotor (Beckman Coulter, Indianapolis, USA) to deplete microvesicles. To pellet sEVs, the same rotor was used to centrifuge samples for 3 h at 100,000× *g*. Collected sEVs were washed with PBS and centrifuged at 100,000× *g* for 1 h and finally resuspended in 200 μL PBS and stored at −80 °C. A Bradford protein assay kit (Thermo Scientific, Rockford, USA, Cat No. 23236) was used to assess sample protein concentrations. The average amount of EV protein from three replicate samples was roughly 22 ± 5, 27 ± 6 and 34 ± 6 μg for MCF10A, MCF7 and MDA-MB-231 cell lines, respectively. These samples were used for proteomic analysis.

### 2.3. Quantification of sEVs by Nanoparticle Tracking Analysis (NTA)

The average particle diameter and concentration of isolated EV samples were measured by the ZetaView PMX-110 (Particle Metrix, Meerbusch, Germany). The camera shutter speeds were adjusted to 85 and 40. The instrument was calibrated and focused with 102 nm polystyrene beads (Microtrac, Cat No. 900383).

### 2.4. Sample Preparation for Phosphoproteomics

Isolated sEVs were resuspended in the lysis buffer with a volume ratio of 4:1 (fraction/buffer) with a final concentration of 20 mM HEPES, pH 8.0, 5% glycerol, 0.1% n-Dodecyl-β-D-Maltoside, 0.2 mM DTT, 1.6 M urea, and 1/250 (v:v) phosphatase inhibitors (phosphatase inhibitor 2 and 3; Sigma: Cat No. P0044 and P5726) and gently vortexed for 3 min. The suspension was centrifuged for 1 min at 10,000× *g,* and the supernatant was then collected.

### 2.5. Phosphopeptide Enrichment by IMAC and TiO_2_

Protein samples obtained from the isolated sEVs were reduced, alkylated, digested, and desalted, as described previously [6]. Phosphopeptide enrichment by IMAC was performed according to the protocol of Pierce Fe-NTA phosphopeptide enrichment kit (Thermo Scientific, Cat No. 88300). TiO_2_-phosphopeptide enrichment was carried out according to the manufacturer’s protocol (Pierce TiO_2_ phosphopeptide enrichment and clean-up kit, Cat No. 88301). All enriched phosphopeptide samples were acidified by adding TFA (1% final concentration), subsequently desalted (TopTip C-18 columns; Glygen Corp.), and dried in a vacuum evaporator.

### 2.6. Nano-LC-MS/MS

To process samples, the Orbitrap Fusion mass spectrometer (Thermo Fisher Scientific, Mississauga, ON, Canada) coupled to an UltiMate 3000 nanoRSLC (Thermo Fisher Scientific, Mississauga, ON, Canada) was utilized, as previously described with modified instrument parameters [6,7]. Following the reconstitution of digested peptides with 20 μL MS-grade H_2_O/1% formic acid (*v*/*v*), three microliters of sample was injected onto an in-house packed column and eluted for 65 min at a flow rate of 300 nL/min (0–10 min, 2–2% ACN; 10–40 min, 2–38% ACN; 40–45 min, 38–98% ACN; 45–50 min, 98–98% ACN; 50–55 min, 98–2% ACN; 55–65 min, 2–2% ACN). The ESI+ parameters were set as follows: top speed mode, ion source temperature 250 °C, ion spray voltage 2.1 kV, and a full-scan MS (m/z 350–2000) resolution of 60,000. For collision-induced dissociation (CID), the automatic gain control (AGC) target was set to 5 × 10^5^ for full scans and 1 × 10^4^ for MS/MS scans. Precursor ions were filtered from +2 to +7 charge states within 2 m/z isolation windows. CID in the linear ion trap was performed at a normalized collision energy of 35%. For the higher-energy collision dissociation (HCD) method, the AGC target for precursor ions was set to 5 × 10^5^ with an ion filling time of 150 ms. The highest intensity ions were isolated and fragmented with a normalized collision energy of 32% and detected at a mass resolution of 15,000. The AGC target for MS/MS was set to 5 × 10^4^ with a maximum injection time of 150 ms and a dynamic exclusion of 30 s.

### 2.7. MS Spectra Processing

MS raw files were analyzed with MaxQuant (version 2.0.1.0) [22] and the built-in Andromeda search engine [23], as previously described with slight modifications [6]. Peptides were searched against the human UniProt FASTA file, containing 20,396 entries (21 April 2021), and a default contaminants database. MaxQuant’s default parameters were used unless otherwise stated. In addition to N-terminal acetylation and methionine oxidation, phosphorylation of serine, threonine, and tyrosine were set as variable modifications. Meanwhile, carbamidomethylation of cysteine was set as a fixed modification. Tryptic peptides with a minimum of 6 amino acids and a molecular weight maximum of 4600 Da were searched with a maximum of two missed cleavages. The initial precursor mass deviation was set to 10 ppm, while a fragment mass deviation of 0.5 Da was used. The false discovery rate (FDR) was set to 0.01 for peptide spectrum matches (PSM) and protein identification, using a reverse sequence decoy database.

### 2.8. Data Filtering and Phosphorylation Site Localization

MaxQuant output tables for protein groups, peptides, and phosphosites were used for all analyses in R [24]. We only retained phosphopeptides, which were measured in at least two out of the three replicates. Remaining phosphopeptides were used to classify phosphosites based on their combined localization probability into class I, II and III, and IV [25]. Known phosphosites and kinase-substrate interactions were downloaded from PhosphoSitePlus (30 July 2021) [26]. In addition, kinases were predicted for remaining phosphosites using GPS 5.0, with the threshold parameter set to high [27]. Predicted kinases were represented as kinase groups and related to previously identified kinases in MDA-MB-231 sEVs [6].

### 2.9. Disease and Functional Annotation Analysis

DisGeNET protein–disease annotations were retrieved with the disgenet2r R package [28]. Cancer- and breast cancer-related proteins were identified by semantically related terms such as “mammary carcinoma” or “malignant neoplasm of breast”. Gene ontology functional annotations were obtained with the org.Hs.eg.db annotation package [29,30,31]. Absolute number of proteins for each biological term was compared between cell lines.

### 2.10. Data Availability

All MS raw data were submitted to the PRIDE repository (Accession: PXD030424) at the European Bioinformatics Institute.

### 2.11. ATP-Citrate Synthase Activity Assay

Cells and sEVs were lysed by gentle vortexing in extraction buffer provided by the ACLY Activity Assay Kit (AMSBIO, Cat No. 79904). Supernatants were collected and used for the analyses of enzymatic activities. ACLY Activity Assay Kit was used according to the manufacturer’s instructions. The assays were performed at room temperature for 60 min.

### 2.12. Phosphofructokinase Activity Assay

The phosphofructokinase activity was measured using kits from Sigma-Aldrich (Phosphofructokinase Activity Colorimetric Assay Kit MAK093) according to manufacturer’s instructions. The assays were performed at room temperature in 100 μL of reaction mixture. The phosphofructokinase activity was calculated via the standard curve of the NADH standard at known concentrations. The samples were mixed and incubated for 30 min, and the absorbance was monitored at 450 nm.

### 2.13. SIRT1 and SIRT6 Activity Assay

The enzymatic activity of SITR1 and SIRT6 was assessed by using the Fluorescent Screening Assay Kit (ab156065, ab156068; Abcam, Toronto, Canada) following the manufacturer’s instructions. The protein extract from cells and EVs was obtained by treatment with Triton X-100 (final concentration 0.5%). The assay was performed in 96-well black microplate (Greiner Bio-One 655209; Fischer Scientific, Monroe, USA) with a reaction volume of 50 μL per well. Briefly, the reaction was started by incubating the protein extract with the reaction mixture containing an acetylated peptide substrate. Samples were incubated for 30 min at 23 °C. Control samples were prepared in absence of NAD^+^. Fluorescence intensities of SIRT1 (λ_ex_ = 340 nm, λ_em_ = 450 nm) and SIRT6 (λ_ex_ = 488 nm, λ_em_ = 530 nm) were measured using a microplate reader (FilterMax F3 and F5 Multi-Mode Microplate Readers from Molecular Devices). The activity of enzymes was calculated from the assay time between 5 and 10 min.

### 2.14. Western Blot

SDS electrophoresis and Western blot experiments were carried out as previously described [6].

## 3. Results

### 3.1. Isolation of sEV

**Figure 1:**
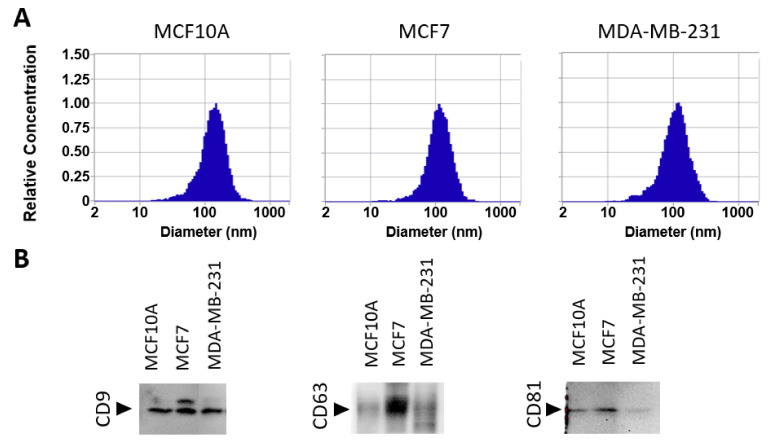
Characterization of extracellular vesicles by nanoparticle tracking analysis (NTA) and Western blot. (**A**) NTA characterization showing size distributions of isolated sEVs from MCF10A, MCF7, and MDA-MB-231 cell lines. (**B**) Western blot analysis of sEV marker proteins, CD9, CD63, and CD81.

In this work, we carried out a systematic analysis of the phosphoproteome of sEVs derived from MCF10A, MCF7, and MDA-MB-231 cell lines to provide insight into the molecular mechanism of breast cancer. To evaluate the range of measured diameters of sEVs isolated by differential ultracentrifugation, nanoparticle tracking analysis was performed. Isolated vesicles, from the three cell lines, measured 50 to 300 nm in diameter, with an average size of about 125 nm (Figure 1A). Additionally, Western blots were performed to validate the presence of EV markers CD9, CD63, and CD81. The results revealed the presence of markers in all free sEV fractions derived from three cell lines: MCF10A, MCF7, and MDA-MB-231 (Figure 1B and Figure S1).

### 3.2. Overall Phosphoproteome Profiling

**Figure 2:**
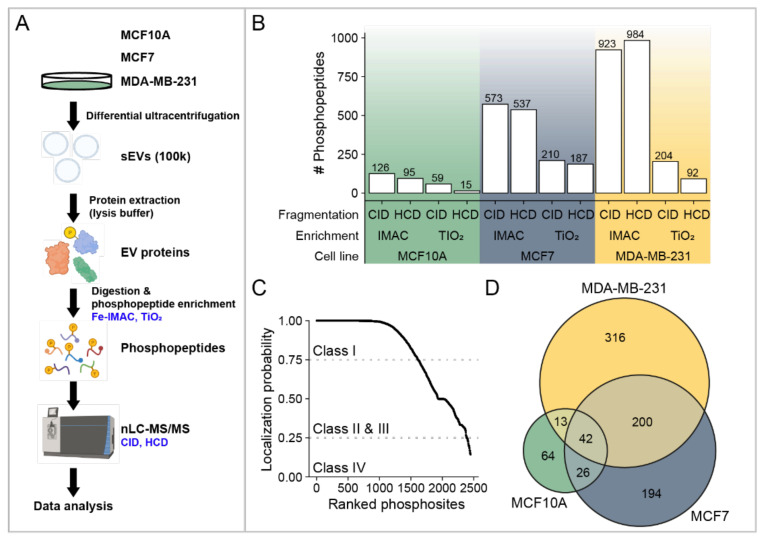
Overview of the identified phosphopeptides and phosphoproteins. (**A**) Workflow illustration of phosphoproteome analysis of sEVs from MCF10A, MCF7, and MDA-MB-231 cells. (**B**) Total number of phosphopeptides identified using Fe-IMAC and TiO_2_ enrichment methods, as well as two fragmentation methods: CID and HCD. (**C**) The probability of correct site identification in peptide sequences. (**D**) A Venn diagram showing the number of EV phosphoproteins identified from the sEVs of three cell lines.

A total of three biological replicates were prepared from MCF10A-, MCF7-, and MDA-MB-231-derived sEVs and examined independently. EVs were buffered with phosphatase inhibitors, digested, and the phosphopeptides were enriched to enhance the identification of low-abundance phosphorylated proteins. In addition, we combined two techniques for the enrichments of phosphopeptides: Fe-IMAC and TiO_2_ affinity chromatography. An overview of our experimental strategy is presented in Figure 2A. The enriched phosphopeptides were analyzed by ultra-high-performance nLC-nESI-MS/MS using two fragmentation modes (CID and HCD), and the obtained data were subjected to rigorous assessment and peptide identification. Each sample represents the data obtained from peptides of three independent experiments after filtering as described in Materials and Methods (Section 2.8, Data Filtering). The reliable phosphopeptides were considered if they were identified in at least two biological replicates. After removal of potential contaminants, the total number of identified phosphopeptides for each sEV sample, using two enrichment and fragmentation methods, are presented in Figure 2B and Table S1. Significant differences were observed using CID and HCD fragmentation for both enrichment methods and Fe-IMAC and TiO_2_ affinity chromatography from all sEVs (Figure S2). In addition, a higher number of phosphopeptides was obtained using IMAC in comparison with TiO_2_. In total, from 2003 phosphopeptides, 207, 854, and 1335 were identified in MCF10A, MCF7, and MDA-MB-231 sEVs, respectively. Among all identified phosphosites (2450), the probability of correct site analysis identified a total of 1613 class I (>75% confidence), 774 class II and III (25–75% confidence), and 63 class IV (<25% confidence) phosphosites, respectively [25]. Among Class I phosphosites, 60 are novel phosphorylated sites, not previously reported (Table S2. Identified phosphopeptides correspond to 145, 462, and 587 phosphoproteins in MCF10A-, MCF7-, and MDA-MB-231-derived sEVs, respectively. The Venn diagram of combined phosphoproteins from three cell lines revealed a total of 855 distinct proteins (Figure 2D).

### 3.3. Phosphorylation Site Distributions, Phosphorylation Motifs and Predicted Potential Kinases for Identified Phosphorylation Sites

**Figure 3:**
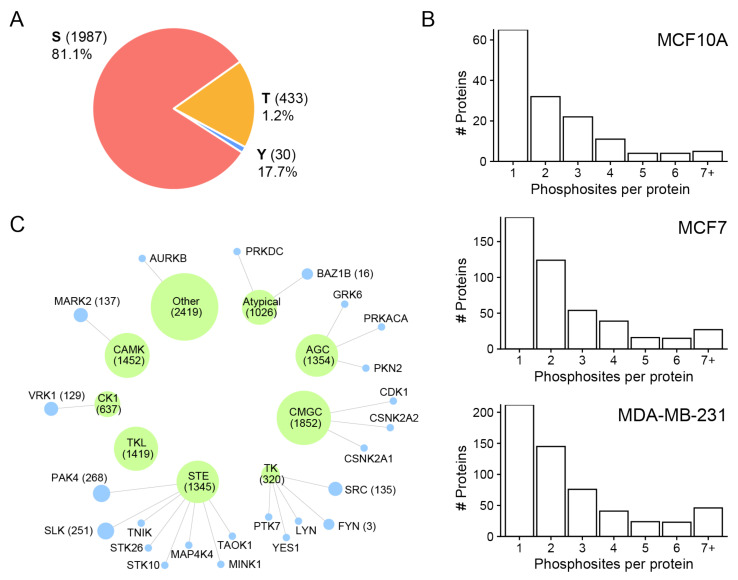
Overview of the identified phosphosites and frequency distribution of phosphorylated amino acid and phosphorylation motifs. (**A**) Frequency distribution of phosphorylated amino acid: pS, pY, and pY. (**B**) Numbers of sites observed per protein in all three sEV fractions. (**C**) Pie chart representation of predicted kinases groups (green) and their link to identified kinases (blue) in MDA-MB-231 sEVs [6]. Node size represents the scaled number of predicted phosphorylation sites.

Figure 3A presents the distribution of phosphosite residues for different cell line-derived sEVs. In total, 1987 phosphoserine (pS), 433 phosphothreonine (pT), and 30 phosphotyrosine (pY) sites were identified (Table S2). Similar distributions of pS, pT, and pY sites were observed among sEVs from the three cell lines. The result revealed that pS was most strongly represented, followed by pT and pY sites. The distribution of the number of phosphorylation sites localized on each protein from the three cell lines is given in Figure 3B. A single phosphorylation site was localized on most of the identified phosphoproteins in all three sEV fractions. An important number of the identified phosphoproteins was found to be phosphorylated more than once. Small EVs derived from MCF7 (275 phosphoproteins; 60.0%) and MDA-MB-231 (355 phosphoproteins; 62.3%) cell lines contained a higher number of multiply phosphorylated proteins than MCF10A (78 phosphoproteins; 54.5%) cells (Figure 3B). Some proteins contained a high number of phosphorylated sites, such as serine/arginine repetitive matrix protein 2 (SRRM2) (72 in MDA-MB-231 EVs, 81 MCF7 EVs, and only 3 in MCF10A). Other proteins that phosphorylated multiple times were mainly present in MDA-MB-231 sEVs, and MCF7 sEVs include Bcl-2-associated transcription factor 1 (BCLAF1), Serine/threonine-protein kinase PRP4 homolog (PRPF4B) and Thyroid hormone receptor-associated protein 3 (THRAP3) (Table S3).

We visualized sequence motifs for the phosphorylated amino acids for each cell line fraction (Figure S3). The majority of phosphorylation sites that contained serine and threonine residues were followed by proline motifs (P at +1). These motifs for pS and pT are well known to be targeted mitogen-activated protein kinases (MAPKs) and cyclin-dependent kinases (CDKs) [32]. Significantly enriched S-based motifs were (D,E+1). The substrate of peptides containing D/E-rich motifs belonged to the casein kinase 1 (CK1) and 2 (CK2) families and some other kinases [33].

In addition, we used the GPS 5.0 software [27] to predict which kinases are responsible for phosphorylation of proteins at the identified sites. Among the phosphorylation sites identified in this analysis, most of them were predicted to be phosphorylated by groups of kinase family such as CMGC, CAMKs, STE, AGC, TKL, CK1, and TK (Figure 3C). These results were consistent with those derived from the phosphosite sequence motifs. Kinases previously identified in MDA-MB-231 EVs [6] mainly belonged to the STE and TK kinase families that largely include subfamily members p21-activated kinase 4 (PAK4), STE20-like serine/threonine-protein kinase (SLK), tyrosine-protein kinase Fyn (FYN), and proto-oncogene tyrosine-protein kinase Src (SRC).

### 3.4. Identified Phosphoproteins in the Context of Cancer/Breast Cancer

**Figure 4:**
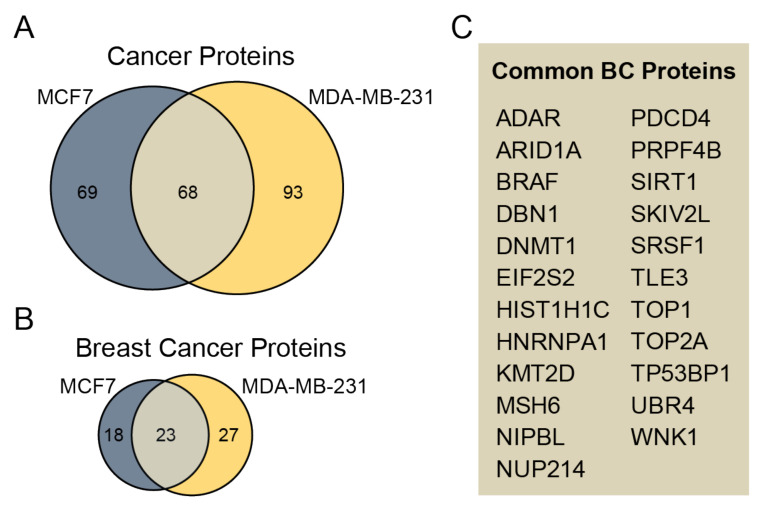
Assessment of identified phosphoproteins related to cancer. (**A**) The Venn diagram compares cancer-related phosphoproteins unique for MCF7 and MDA-MB-231 sEVs. (**B**) The Venn diagram compares the number of phosphoproteins relevant to BC unique for sEVs from cancerous cell lines. (**C**) A list of 23 common BC proteins from MCF7 and MDA-MB-231 sEVs is presented.

To determine whether identified phosphoproteins are linked to cancer diseases, we annotated proteins using the recent set of disease annotations from the DisGeNET database [28]. Phosphoproteins are associated with several diseases, but they are most commonly related to cancer (Figure S4). In total, 137 and 161 phosphoproteins were associated with cancer diseases in sEVs derived from MCF7 and MDA-MB-231 cell lines, respectively (Figure 4A). Among them, 40 and 50 sEV phosphoproteins derived from MCF7 and MDA-MB-231 are related to breast cancer, respectively (Figure 4B). The commonly associated breast cancer phosphoproteins unique to both MCF7 and MDA-MB-231 sEVs include 23 phosphoproteins (Figure 4C, Table S3). Furthermore, phosphosites unique for MCF7 and MDA-MB-231 sEVs were compared with previously identified phosphosites in plasma EVs in patients diagnosed with breast cancer and healthy controls [18]. We found in our study nine phosphosites that were common with previously reported studies (Table S4).

### 3.5. Functional and Pathway Analysis of Identified Phosphoproteins

**Figure 5:**
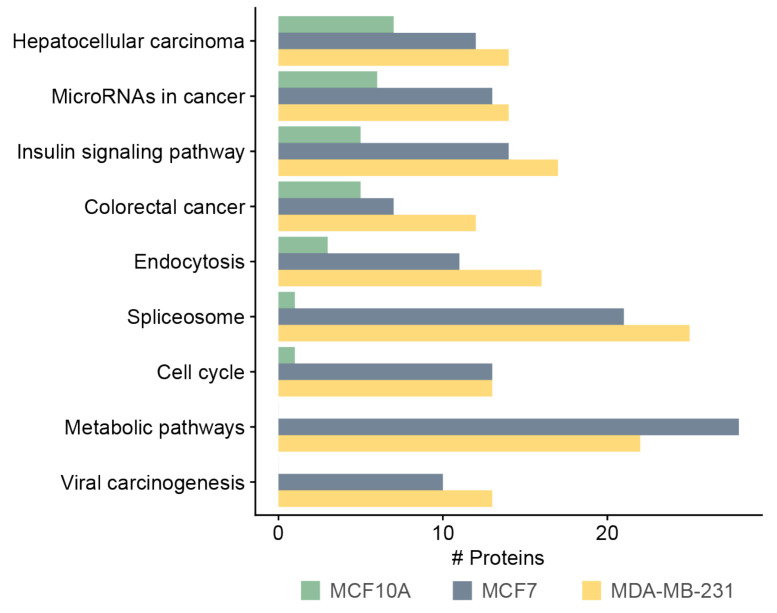
KEGG pathway analysis of phosphoproteins derived from MCF10A, MCF7, and MDA-MB-231 sEVs.

We annotated and classified proteins using the KEGG pathway database. Pathways terms such as metabolic pathway, spliceosome, cell cycle, and viral carcinogenesis were highly abundant in MCF7- and MDA-MB-231-derived sEVs, compared with MCF10A-derived sEVs (Figure 5) (Table S5). Interestingly, 13 and 10 proteins identified in the MDA-MB-231 and MCF7 sEVs, respectively, were related to cell cycle. Among these cell cycle proteins, five proteins belonged to kinases: cyclin-dependent kinase 1 (CDK1) and 2 (CDK2), glycogen synthase kinase 3 beta (GSK3B), polo-like kinase 1 (PLK1), and protein kinase/DNA-activated, catalytic subunit (PRKDC) (Table S5). We previously studied the presence of three functional metabolic enzymes in BC-derived MVs that might be used as potential biomarkers in BC therapy [7]. This investigation indicated that sEV enzymes can effectively be incorporated into accurate, quick, and sensitive early diagnostic assays that work by measuring their enzymatic activities. Therefore, we focused on sEV enzymes annotated by the term “metabolic pathways” by the KEGG database. After data analysis, six enzymes were identified only in the MCF7- or MDA-MB-231-derived sEVs at least once in three biological replicates. These enzymes are involved in the biosynthesis of cofactors (ACLY, ACSS2), glycolysis (PKFM), deacetylase activity (SIRT1 and SIRT6), and pyrimidine biosynthesis (CTPS1) (Table 1). Two enzymes, ACLY and PKFM, contained the highest number of identifications of phosphopeptides among the list of proteins presented in Table 1. The SIRT1 and SITR6 enzymes were identified in all three biological replicates of MCF7 and/or MDA-MB-231 sEVs with the IMAC phosphopeptide enrichment technique using either CID or HCD fragmentation methods. Therefore, four enzymes that have at least six identifications of phosphopeptides were further investigated: ACLY, PKFM, SIRT1, and SIRT6. Representative MS/MS spectra for these four enzymes are presented in Figure S5. Measuring the enzymatic assay for CTPS1 requires specific instrumentation, is time consuming, and is difficult to implement for routine use [34]. For this reason, no further validation of this enzyme was performed.

### 3.6. Analysis of ACLY, PKFM, SIRT1, and SIRT6 in Cells and Their sEVs

**Figure 6:**
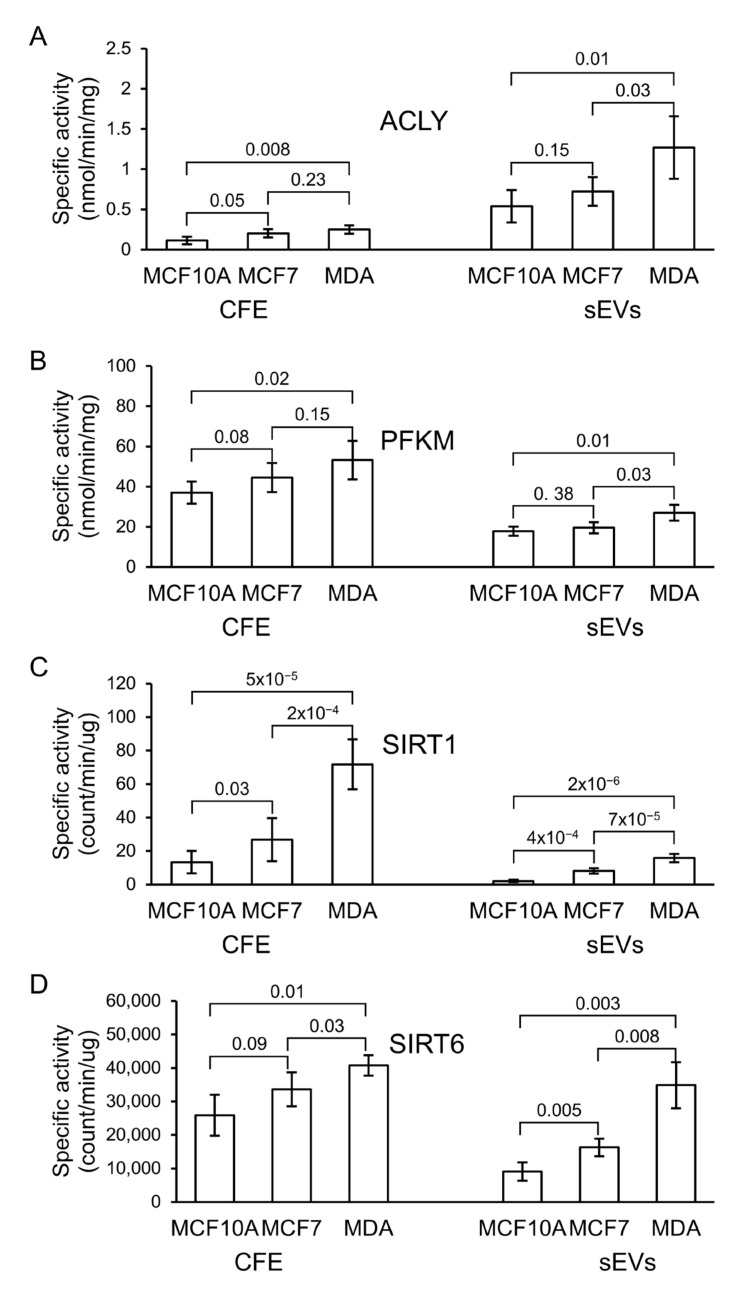
Specific enzymatic activity of (**A**) ATP citrate lyase (ACLY), (**B**) phosphofructokinase-M (PFKM), (**C**) sirtuin-1 (SIRT1), and (**D**) sirtuin-6 (SIRT6) in cell-free extract (CFE) and their corresponding sEV fractions. The bar graph represents mean values, while error bars indicate the standard deviation (SD) of four replicates and *p*-values obtained from a Student’s *t*-test.

ACLY, PKFM, SIRT1, and SIRT6 activities were detected in all extracted protein fractions from the three cell lines, as well as their sEVs (Figure 6, Table S6). The specific activity of ACLY was significantly higher in MDA-MB-231 when compared with MCF7- and MCF10A-derived sEVs. The specific activity of PFKM in sEV fractions, although slightly higher in MDA-MB-231 in comparison with the other two cell lines, was relatively similar in all sEV fractions. The specific activity of SIRT1 and SIRT6 in MDA-MB-231 and MCF7 was substantially higher in comparison with MCF10A in both cell-free extract (CFE) and sEV fractions, respectively (Figure 6). However, a more significant difference in specific activity between sEVs derived from non-cancerous and cancerous cell lines was observed for SIRT1.

## 4. Discussion

To understand the molecular and cellular regulatory mechanisms important for BC tumor progression and metastasis, we performed a phosphoproteomic analysis of BC-derived sEVs. In our study, we used sEVs from two breast cancer cell lines, MCF7 and MDA-MB-231, and a non-tumorigenic breast cell line, MCF10A. Two enrichment techniques, IMAC and TiO_2_, and fragmentation methods, CID and HCD, were employed to maximize the purification and identification of phosphopeptides. We identified 2450 phosphorylation sites with 60 novel ones. These phosphorylation sites were mapped to 870 distinct proteins, covering a broad range of functions. Many identified phosphoproteins were unique to BC EVs encompassing a variety of signaling, metabolic, and regulatory pathways and cellular processes. The significant difference in the number of phosphoproteins identified from sEVs isolated from MCF10A and either MDA-MB-231 or MCF7 could be due to MCF10A producing a lower number of sEVs. This is supported by studies showing that MCF10A cells produce significantly fewer sEVs and less protein content than MDA-MB-231 [6,35]. Moreover, differences in the phosphoproteins identified were also observed between MCF7 and MDA-MB-231 EVs (Figure 2D). Furthermore, the intensity of EV markers observed by Western blot analysis varied for these cell lines, particularly for CD63 and CD81 markers (Figure 1B). The difference in phosphoproteome patterns and abundance of EV markers may be attributed to natural cell-to-cell variation in protein expression and sEV biogenesis. Distinct cell lines may release different ratios of EV subtypes that consequently change the overall number of identified proteins, as well as their expression.

In this study, among all groups of kinases, CMGC, CAMKs, STE, AGC, and TKL were predicted to regulate the largest number of phosphorylation events in sEVs (Figure 3C). Kinases previously identified in MDA-MB-231 EVs [6] mainly belong to the STE and TK kinase families including subfamily members such as p21-activated kinase 4 (PAK4), STE20-like serine/threonine-protein kinase (SLK), tyrosine-protein kinase Fyn (FYN), and proto-oncogene tyrosine-protein kinase Src (SRC). The PAK4 protein kinase is often highly expressed in TNBC cells and plays an important role in cell growth, survival, and migration [36]. The Ste20-like kinase, SLK, is involved in the control of BC cell motility [37]. It has been reported that FYN, a member of the SRC family kinases, is required for the maintenance of the basal breast cancer subtype [38]. For instance, c-Src proto-oncogene tyrosine kinase has been shown to support cancer cell migration and proliferation [39]. The presence of these kinases in EVs may be important for the regulation of protein phosphorylation in recipient cells, and consequently, the promotion of cancer progression.

In total, 266 phosphoproteins were associated with cancer in sEVs derived from MCF7 and MDA-MB-231 cell lines. Among them, 78 are related to breast cancer, suggesting that the cargo of phosphoproteins in sEVs may play an important role in cancer progression and metastasis. Moreover, these identified phosphoproteins could serve as biomarkers for BC diagnosis. Therefore, this study identified nine proteins (Table S4) previously found in plasma EVs of patients diagnosed with BC and healthy controls [18]. These nine phosphoproteins may be further explored in BC biomarker development and clinical use.

Advancements in proteomics facilitate discovery of enzymes that can provide an attractive source for cancer biomarker tests. Due to their unique enzyme specificity and selectivity, enzymatic assays are a reliable, simple, and rapid diagnostic method [40]. A phosphoproteomic approach is particularly important because the phosphorylation of enzymes may affect enzymatic activities [14,41,42,43]. Therefore, for the selection of potential BC biomarkers for detection and prognosis as well as for pharmacological purposes, the focus in this work was on the identification of phosphorylated sEV enzymes identified only in BC cell lines. This led to the selection of phosphorylated enzymes present only in cancerous cell lines. These enzymes include ACLY, PKFM, SIRT1, and SIRT6.

ACLY is a cytoplasmic homologous tetramer composed of four polypeptide chains and acts as a metabolic enzyme involved in fatty acid synthesis in rapidly proliferating cancer cells [44,45,46]. ACLY plays a role in modulating proliferation, growth, migration, and apoptosis that has been reported in many cancer cells [44,45,46]. The expression of ACLY in human lung adenocarcinoma has been investigated to be higher compared with non-carcinogenic lung control tissue [47] and contributes to increased lipogenesis and tumor growth [48]. It has been shown that miR-22 inhibits the growth and metastasis of MCF7 cells by decreasing ACLY expression [49]. BMS-303141, an ACLY inhibitor, has been suggested for HCC treatment [50]. It could induce endoplasmatic reticulum stress and activate the p-eIF2α/ATF4/CHOP axis, promoting HCC cell apoptosis. Recent studies highlight ACLY as a potential biomarker for predicting breast cancer recurrence in patients [51]. In this study, we found that ACLY enzymatic activity was increased in breast cancer-derived sEVs isolated from the MDA-MB-231 cell line, in comparison with sEVs isolated from MCF7 and MCF10A cell lines. Further studies are needed to investigate the potential of this protein as a clinical indicator for prognosis in breast cancer.

Expression levels of phosphofructokinase-M (PFKM) are closely related to the occurrence and development of malignant tumors. To meet the metabolic demands of tumor cells in energy, the activity of PFKM in cancer cells is increased [52,53,54,55,56]. The inhibition of 2,6-2-fructose production decreases PFKM activity, which results in the inhibition of the growth of tumor cells [57]. The gene-based analysis of early-onset BC has identified a region containing the key glycolysis regulation gene, PFKM, which is proposed as a potential target for BC prevention and treatment [58]. Our study indicates that the specific activity of PFKM in sEV fractions of MDA-MB-231 was slightly higher than its activity in sEVs from MCF7 and non-cancerous MCF10A cell lines, suggesting that the phosphorylation of PFKM in MDA-MB-231 sEVs does not significantly affect the enzyme’s activity. Therefore, this enzyme from sEVs might not effectively serve as a prognostic biomarker for BC.

Sirtuin-1 (SIRT1) is a class-III histone deacetylase (HDAC) enzyme involved in gene regulation, genome stability, apoptosis, autophagy, senescence, proliferation, aging, and tumorigenesis [59]. This enzyme deacetylates histones and non-histone proteins important in cancer biology such as p53, p73, Rb, and NF-κB [60,61]. SIRT1 is proposed as a prognostic indicator as well as a novel therapeutic candidate in triple-negative breast cancer (TNBC) [62]. Studies looking at the expression of SIRT1 in BC indicate its contradictory roles as a tumor suppressor or promoter [63,64,65]. Specific activity of SIRT1 from whole cell and sEV protein fractions is higher in MCF7 and MDA-MB-231 than its corresponding MCF10A fractions. This suggests that SIRT1 could be useful to investigate as a potential prognostic and therapeutic target for BC.

Sirtuin-6 (SIRT6) is involved in multiple molecular pathways related to DNA repair, glycolysis, gluconeogenesis, tumorigenesis, neurodegeneration, and cardiac hypertrophic responses [66]. SIRT6 may be linked to cancer progression and tumor growth. It was identified as a tumor suppressor that regulates aerobic glycolysis in cancer cells [67,68]. In vivo studies revealed the critical role of high SIRT6 levels in slowing down hepatic cancer at an early stage of its progression [66]. 4H-Chromen, an activator of SIRT6, has been studied in various breast cancer cells and demonstrated to decrease cell proliferation in TNBC cells [69]. In this work, like SIRT1, cancerous MCF7 and MDA-MB-231 cell line-derived sEVs exhibited higher enzymatic activity of SIRT6 than sEVs of the non-cancerous MCF10A cell line. This finding suggests that this enzyme may be useful to investigate as a potential biomarker for BC diagnosis.

Previous studies have investigated the phosphoproteome of BC cell lines, as well as their EVs [70,71]. However, our methodological approach for studying phosphoproteins of BC cell lines was different from these reports [71,72]. Phosphoproteomic analysis of EVs derived from several BC cell lines including MCF7, MDA-MB-231, and MCF10A was performed using only IMAC enrichment and HCD fragmentation methods; ACLY and SIRT1 were identified, but not PFKM and SIRT6. In another report that studied the phosphoproteomic characterization of KAIMRC1, MCF-7, and MDA-MB-231 BC cell lines, TiO_2_ enrichment and CID fragmentation methods were employed for peptide enrichment and fragmentation; the study was based on cell culture lysates and did not identify the four enzymes presented in our work.

The objective of this study, along with previously published works [6,7], was to investigate proteins or enzymes that may be later explored for their efficacy as biomarkers for BC early detection. To achieve this objective, blood sEVs from healthy and BC patients should be studied for the presence or activity of these potential biomarkers. This study was limited by the isolation method of sEVs since extracellular vesicle subtypes are diverse in their diameter and density. Therefore, ultracentrifugation does not differentiate sEVs that originate from different biogenesis pathways. Due to the heterogeneity of EV populations that come from different intracellular origins, our study did not examine a specific EV subtype, but instead considered a diverse population of sEVs. It has been reported that the EV isolation method can significantly impact EV yield and purity from human serum [72]. For this reason, it will be most challenging to determine the most suitable method for the isolation of EVs from blood in the future. In this study, we found that phosphorylated enzymes identified from sEV fractions were already proposed to play a role in cancer therapy. Our findings support further efforts to investigate these enzymes, especially ACLY, SIRT1, and SIRT6 for breast cancer diagnosis and therapy.

## 5. Conclusions

In this study, we conducted a phosphoproteomic analysis of breast cancer-derived extracellular vesicles from MCF10A, MCF7, and MDA-MB-231 cell lines. In total, 855 distinct phosphoproteins were identified collectively among the cell lines, covering a wide range of functions, most of which are related to cancer. Among these phosphoproteins, we validated four enzymes: ACLY, PFKM, SIRT1, SIRT6. The results demonstrate that the specific activity of PFKM in BC cancer cell lines was not statistically different from the non-cancerous cell line. In contrast, the three phosphorylated enzymes ACLY, SIRT1, and SIRT6 showed a significantly higher specific enzymatic activity in MDA-MB-231 in comparison to MCF10A-derived sEVs. These three enzymes might serve as a potential prognostic biomarker for BC. They have been previously proposed as therapeutic targets for cancer therapy. Thus, our findings justify further investigation of these enzymes as promising drug targets for BC treatment.

## Figures and Tables

**Table 1:** Summary of the identifications of phosphosites for enzymes in three biological replicates of MCF7 and MDA-MB-231 sEVs. Six proteins were identified using IMAC and either CID or HCD fragmentation method in all three replicate samples in at least one cell line. In addition, PKFM has also been identified from TiO_2_ enrichment in two replicates using HCD fragmentation in contrast with the rest.

Enzyme	Gene	Number of Phosphorpeptide Identifications	MCF7 sEV		MDA-MB-231 sEV	
			CID	HCD	CID	HCD
			Found in sample replicates (Number/3)			
ATP citrate lyase	ACLY	8	3	3	1	1
6-Phosphofructokinase	PFKM	8	1	0	3	3
Sirtuin 1	SIRT1	7	3	1	0	3
CTP synthetase 1	CTPS1	7	1	0	3	3
Sirtuin 6	SIRT6	6	0	3	0	3
Acetyl-CoA synthetase 2	ACSS2	5	2	3	0	0

## Data Availability

The mass spectrometry proteomics data have been deposited to the ProteomeXchange Consortium via the PRIDE partner repository with the dataset identifier PXD030424.

## Supplementary Materials

The following supporting information can be downloaded at: https://www.mdpi.com/article/10.3390/biomedicines10020408/s1, Figure S1: Full-length images of Western blot gels, Figure S2: Venn diagrams presenting the overlap of phosphopeptides identified from either IMAC or TiO2 enrichment method using two fragmentation methods, CID and HCD, Figure S3: Phosphorylation motifs of EVs from MCF10A, MCF7, and MDA-MB-231 cells for pS, pY, and pY, Figure S4: Fractions of disease annotations for selected disease categories using phosphoproteins unique for MCF7 and MDA-MB-231 sEVs, Figure S5: Representative MS/MS spectra of phosphopeptides from ACLY, PFKM, SIRT1, and SIRT6, Table S1: Total number of identified phosphopeptides for each sEV sample, using two enrichment and fragmentation methods, Table S2: The probability of correct site analysis for phosphoserine, phosphothreonine, and phosphotyrosine sites identified from three cell line-derived sEVs, Table S3: The list of identified phosphoproteins indicating breast cancer phosphoproteins common to MDA-MB-231 and MCF7 sEVs and number of identified phosphosites per protein per cell line, Table S4: Phosphosites found in MCF7 and/or MDA-MB-231 EVs common to previously identified phosphosites in plasma EV of healthy patients and diagnosed with breast cancer [18], Table S5: KEGG pathway functional annotation terms for sEV MCF10A of MCF7 and MDA-MB-231, Table S6: Activities of ACLY, PFKM, SIRT1, and SIRT6 in the different protein extracts from cells and their derived sEV fractions. The numbers in parentheses indicate the number of biological replicates.
