# Supplementary material for: Phosphoproteomic Analysis of Breast Cancer-Derived Small Extracellular Vesicles Reveals Disease-Specific Phosphorylated Enzymes
Source: Biomedicines. 2022 Feb 9;10(2):408. doi: 10.3390/biomedicines10020408 (PMC8962341; doi:10.3390/biomedicines10020408)
Supplement: Supplementary file 1 [file biomedicines-10-00408-s001.zip › Supplemental_Figures_S1-S5.pptx]

## Slide 1
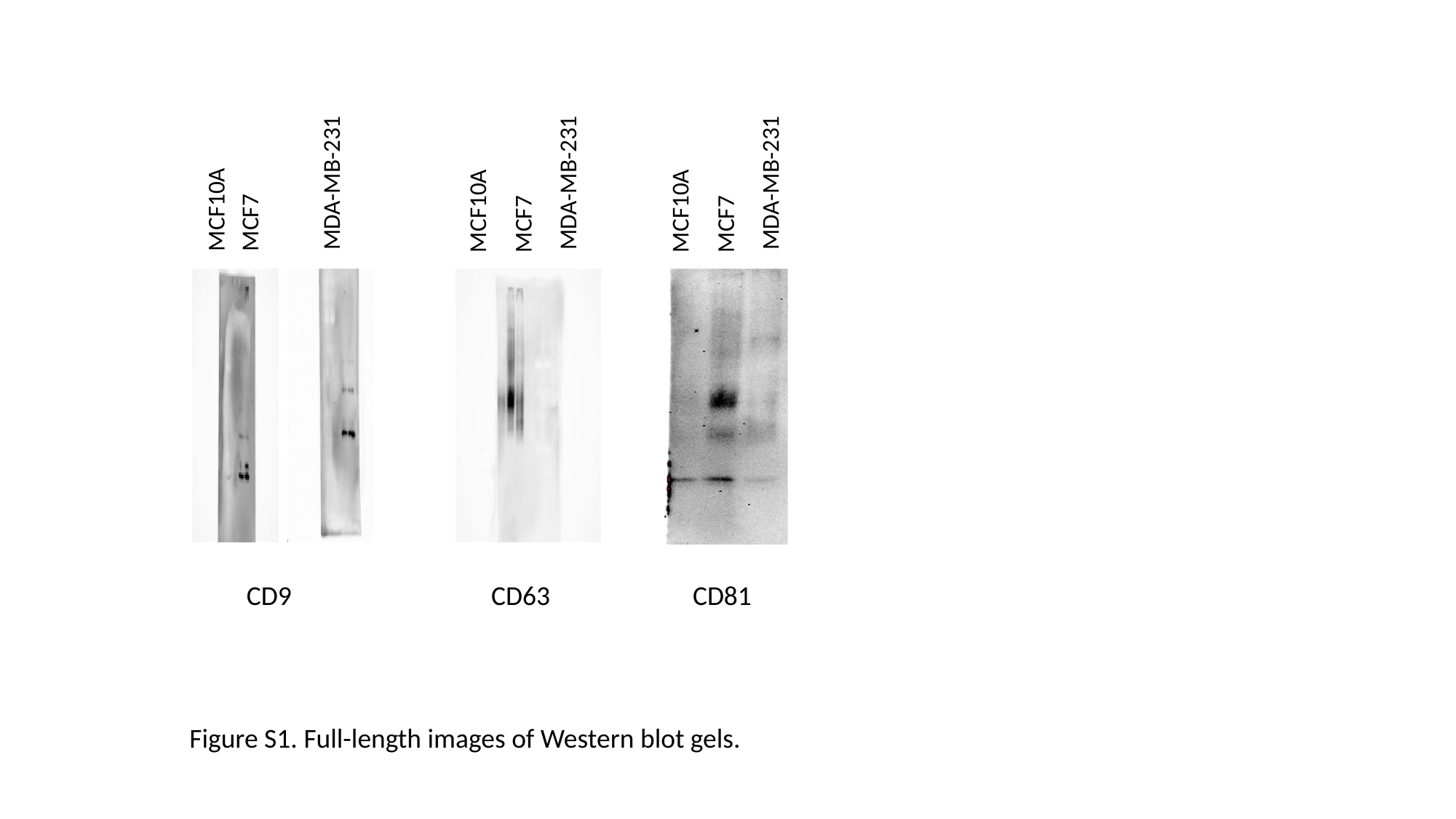

MDA-MB-231
MDA-MB-231
MDA-MB-231
MCF10A
MCF10A
MCF10A
MCF7
MCF7
MCF7
CD9
CD63
CD81
Figure S1. Full-length images of Western blot gels.

## Slide 2
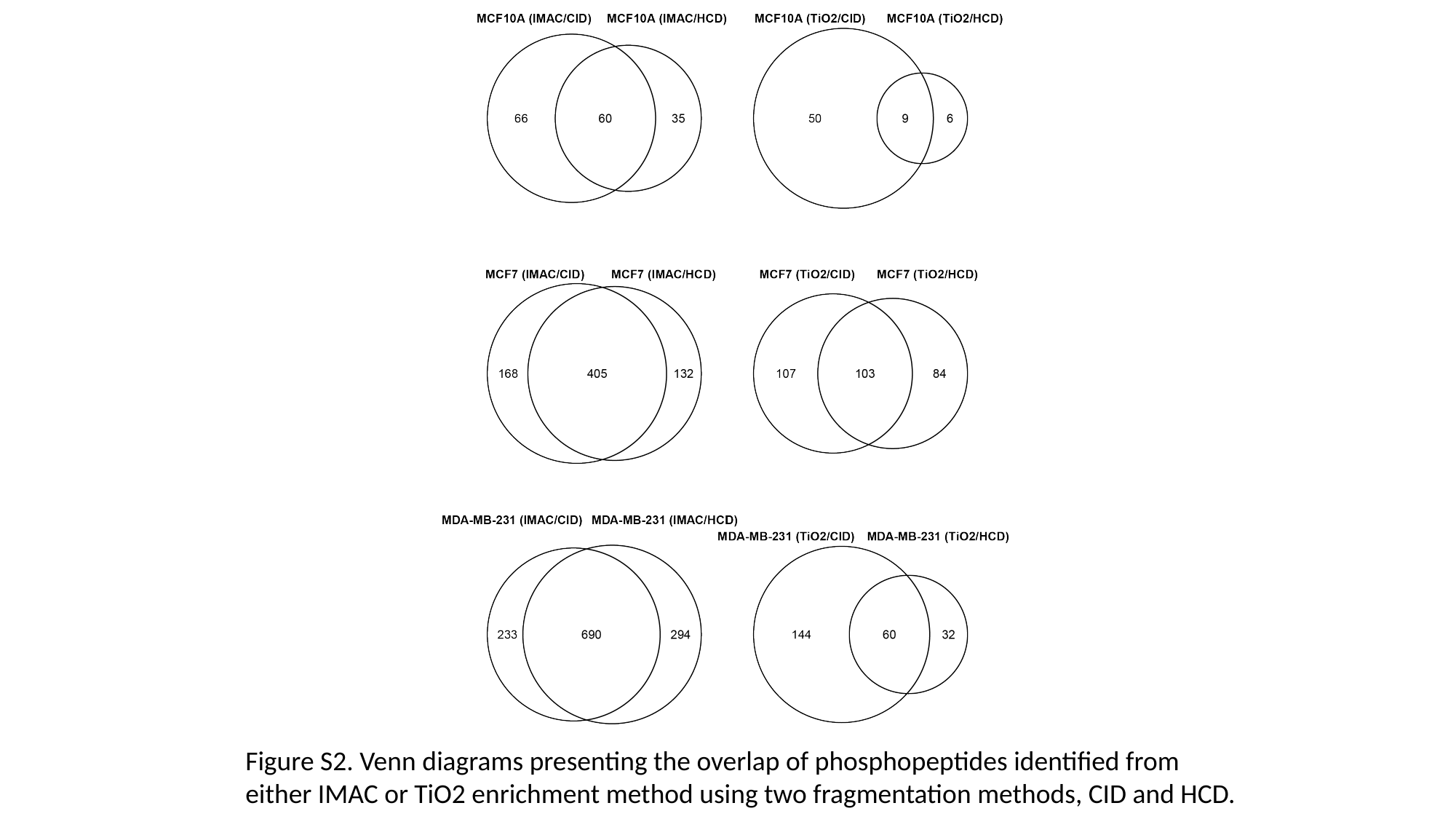

Figure S2. Venn diagrams presenting the overlap of phosphopeptides identified from either IMAC or TiO2 enrichment method using two fragmentation methods, CID and HCD.

## Slide 3
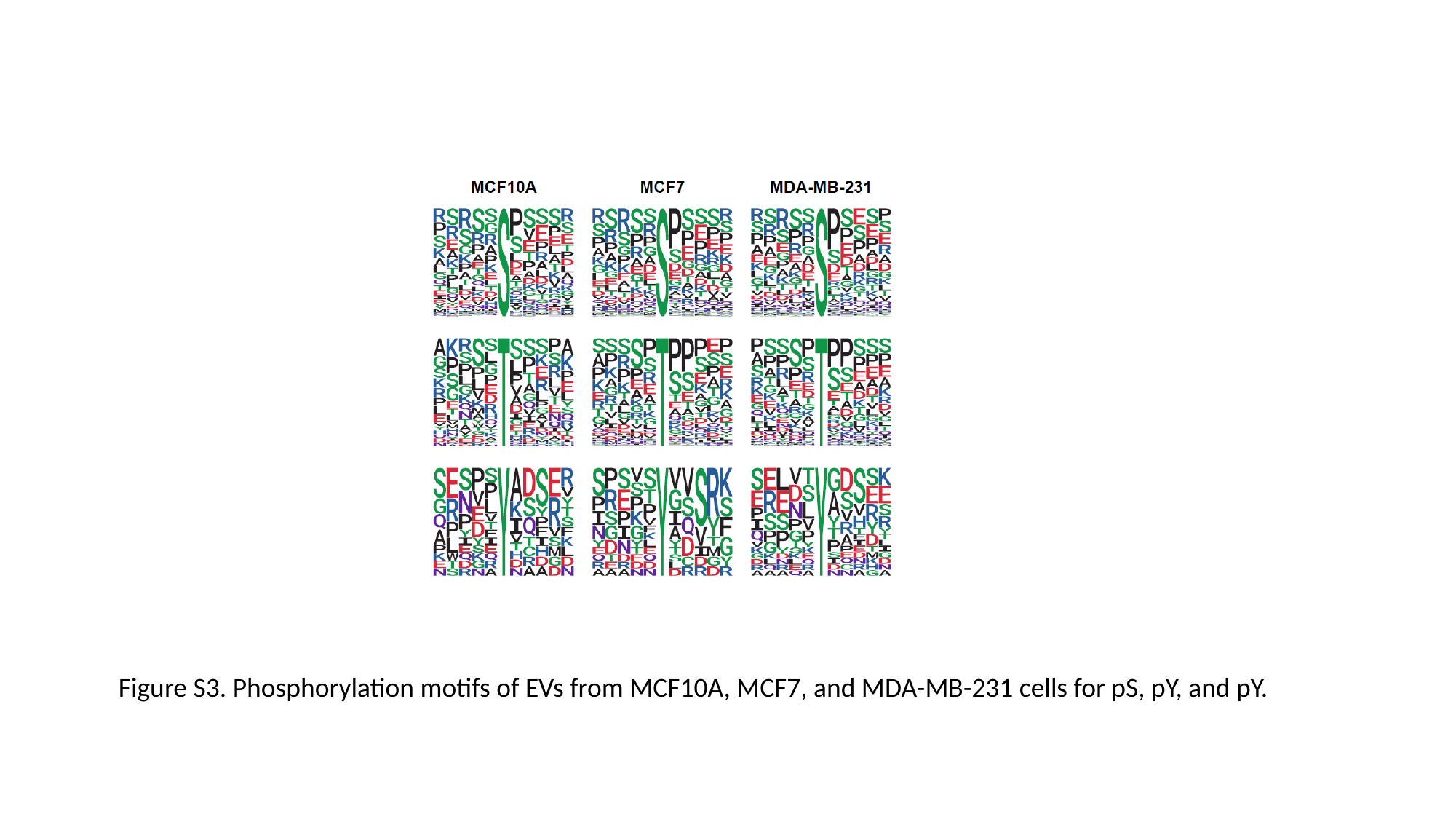

Figure S3. Phosphorylation motifs of EVs from MCF10A, MCF7, and MDA-MB-231 cells for pS, pY, and pY.

## Slide 4
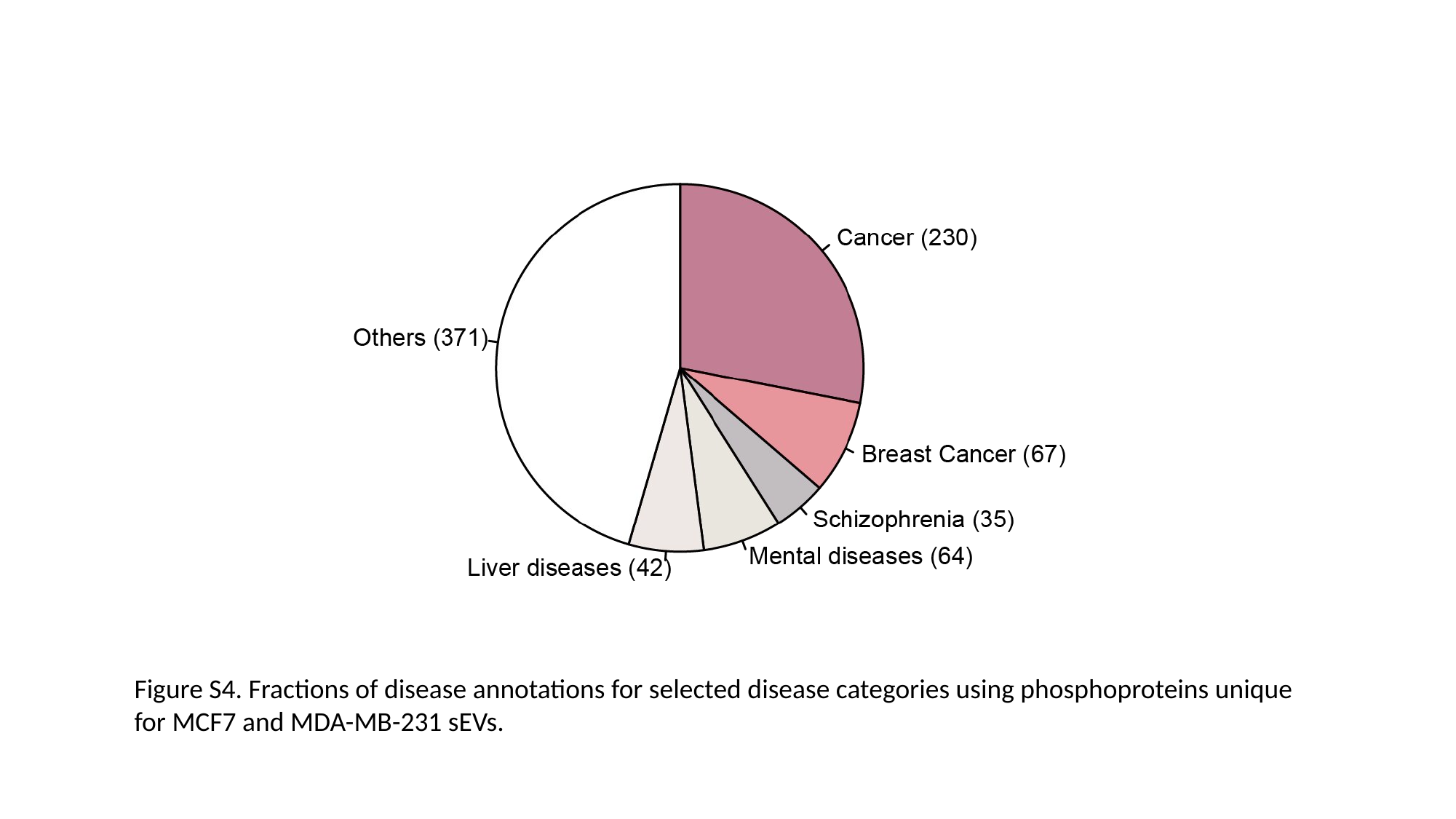

Figure S4. Fractions of disease annotations for selected disease categories using phosphoproteins unique for MCF7 and MDA-MB-231 sEVs.

## Slide 5
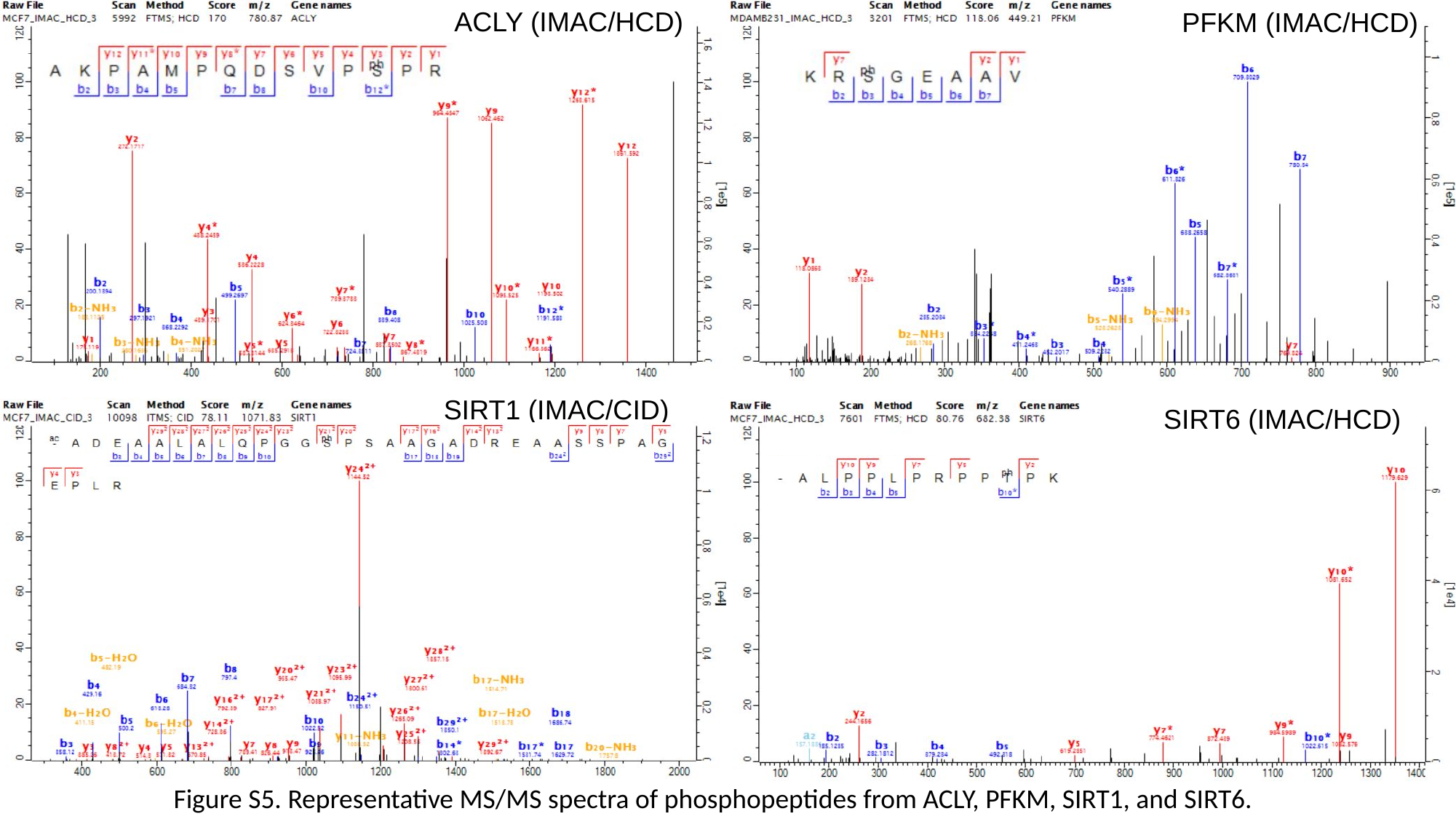

PFKM (IMAC/HCD)
ACLY (IMAC/HCD)
SIRT1 (IMAC/CID)
SIRT6 (IMAC/HCD)
Figure S5. Representative MS/MS spectra of phosphopeptides from ACLY, PFKM, SIRT1, and SIRT6.
